# The correlation of aromatase activity and obesity in women with or without polycystic ovary syndrome

**DOI:** 10.1186/s13048-015-0139-1

**Published:** 2015-03-22

**Authors:** Jie Chen, Shanmei Shen, Yong Tan, Dong Xia, Yanjie Xia, Yunxia Cao, Wenjun Wang, Xiaoke Wu, Hongwei Wang, Long Yi, Qian Gao, Yong Wang

**Affiliations:** First Clinical Medicine College, Nanjing University of Chinese Medicine, Nanjing, 210046 China; State Key Laboratory of Chemistry for Life Science and Jiangsu Key Laboratory of Molecular Medicine, Medical School of Nanjing University, Nanjing, 210093 China; Divisions of Endocrinology, the Affiliated Drum Tower Hospital, Medical School, Nanjing University, Nanjing, 210093 China; Department of Obstetrics and Gynecology, Anhui Medical University, Hefei, 230022 China; Centre of Reproduction, Department of Obstetric and Gynaecology, Memorial Hospital of Sun Yat-Sen University, Guangzhou, 510120 China; Department of Obstetrics and Gynecology, the First Affiliated Hospital, Heilongjiang University of Chinese Medicine, Harbin, 150040 China

**Keywords:** PCOS, Aromatase activity, Obesity, Estradiol, Testosterone

## Abstract

**Background:**

This study aimed to investigate the effect of polycystic ovary syndrome (PCOS) on the association of aromatase activity assessed by estradiol-to-testosterone ratio (E_2_/T) with body mass index (BMI) in women.

**Methods:**

This was a cohort study in five centers for reproductive medicine in China. Data were collected from July 2012 to December 2013. PCOS patients (n = 785) and non PCOS, healthy, age-matched controls (n = 297) were included. Plasma sex hormones including estradiol (E_2_), testosterone (T), follicle stimulating hormone (FSH), and luteinizing hormone (LH) were measured by ELISA, together with BMI and E_2_/T being calculated, on the third day of the menstrual cycle. Aromatase activity in PCOS patients with different BMI, T and E_2_ levels were compared.

**Results:**

E_2_/T was significantly lower (P < 0.05) while BMI was significantly increased (P < 0.05) in PCOS than non-PCOS. No significant difference was observed in E_2_/T among different BMI subgroups of either PCOS or control. Ovarian aromatase activity was decreased in PCOS patients which was independent of BMI. Hyperestrogen promoted ovarian aromatase activity, while hyperandrogen inhibited such activity, both in a dose-dependent, biphasic manner.

**Conclusions:**

Ovarian aromatase activity was lower in PCOS, which was independent of BMI. New therapeutic strategies can be developed by targeting aromatase activity for treating PCOS women, especially those with obesity.

## Background

Polycystic ovary syndrome (PCOS) is a heterogeneous disorder characterized by dysfunction of gonadal axis and systemic nerve endocrine metabolic network [1], with a prevalence of up to 10% in women of reproductive age [2,3]. Furthermore, this number may underestimate the severity of the situation as many women with PCOS in the community remain undiagnosed [4]. PCOS has significant and diverse clinical implications including reproductive, endocrine and metabolic abnormalities such as hyperandrogenism and obesity [3]. Obesity, particularly abdominal obesity, is one of the independent factors aggravating the PCOS endocrine disorders, as subcutaneous abdominal adipose tissues and the liver tissues contribute to extragonadal aromatization [5].

Aromatase, a product of the CYP19 gene [6], is a member of the cytochrome P450 family [7]. Aromatase is a rate-limiting enzyme that catalyzes the conversion of androgens (androstenedione and testosterone) to estrogens (estrone and estradiol) during steroidogenesis [8]. In ovaries, estradiol is generated by converting C19 androgens derived from theca cells under the influence of aromatase produced by granulosa cells [9]. Consequently, the ratio of estradiol (E_2_) to testosterone (T) has been used to evaluate aromatase activity [10,11]. Multiple studies have reported a dysfunctional P450-aromatase activity in PCOS women. However, whether the abnormality is caused by hyperfunction or insufficiency of the enzyme remains unknown [12-16]. The nature of the interaction between ovarian aromatase activity and PCOS in women has been controversial, and the impact of weight gain on aromatase activity as well as E_2_ levels is unknown.

The objective of this study was to investigate the association and interaction between aromatase activity and levels of body mass index (BMI) from a reproductive hormone perspective in a group of women with or without PCOS.

## Methods

### Case origin

We designed a cohort study which included 1082 individuals from five clinical centers (785 PCOS and 297 age-matched non-PCOS) from July 2012 to December 2013. The study was approved by the Medical Ethics Committee of the Medical School of Nanjing University, Nanjing, China.

### Inclusion and exclusion criteria

PCOS was diagnosed according to the 2006 Rotterdam criteria [17]. PCOS may be confirmed if any two out of the following three criteria are met and any other diseases that cause anovulation or hyperandrogenism can be excluded: (1) Oligovulation or anovulation, (2) Clinical manifestation or biochemical evidence of hyperandrogenism, (3) Occurrence of PCO (at least 12 antral follicles measuring 2–9 mm in diameter or the enlargement of an ovarian volume to more than 10 ml by transvaginal ultrasound). The non-PCOS women were selected from infertile couples if the infertility was attributed to male factors in the study period. All the subjects were between 20 and 35 years of age who had not been taking hormone drugs such as contraceptives, ovulation drugs, corticosteroids three months prior to inclusion and who did not have serious heart, liver, renal, and hematopoietic system diseases or malignant tumors.

Controls were recruited from healthy women with a regular menstrual cycle, normal basal sex hormones levels and absence of PCO on sonography.

### Clinical and hormonal analyses

BMI was calculated as weight in kilograms divided by the square of height in metres (kg/m^2^). Peripheral blood samples were taken between 08:00–09:00 A.M. on the third day of the menstrual cycle from all subjects after overnight fasting and frozen at −80°C until assayed. Sex hormones including E_2_, T, luteinizing hormone (LH) and follicle-stimulating hormone (FSH) were measured by ELISA (Beijing North Institute of Biological Technology of China and the CIS Company of France). Intra- and inter-assay coefficients of variation were 10% for all the assays.

### Grouping

Both the PCOS patients and non-PCOS subjects were allocated to one of the three subgroups, namely the obese subgroup (BMI ≥ 23 kg/m^2^), the normal-weight subgroup (18.5 kg/m^2^ ≤ BMI < 23 kg/m^2^) and the underweight subgroup (BMI < 18.5 kg/m^2^), based on WHO recommendations for the Asia-Pacific region [18].

PCOS patients were also divided into subgroups based on the levels of T (T ≥ 2.44 nmol/L or T < 2.44 nmol/L) and E_2_ levels (E_2_ > 293.6 pmol/L, 146.8 ≤ E_2_ ≤ 293.6 pmol/L, or E_2_ < 146.8 pmol/L). The cuts-off were defined by normal laboratory reference values of reproductive medicine centers.

### Statistics

SAS version 9.0 (USA) software was used to match cases and controls based on age. SPSS version 17 (SPSS, Chicago, IL, USA) was used to process the data. Parameters were described using mean ± standard deviation, or median ± quartiles (for data not normally distributed) and the statistical analyses were carried out by t-test and the rank-sum test, respectively. Subgroup differences were calculated by single-factor ANOVA. P < 0.05 was considered statistically significant.

## Results

### Aromatase activity in PCOS

The base sex hormone differences between the PCOS and non-PCOS subjects are summarized in Table 1. PCOS patients showed significantly increased levels of BMI, E_2_, T and LH, while their E_2_/T, FSH and FSH/LH values were decreased compared with the non-PCOS group.

**Table 1:** **Biochemical data from PCOS and non-PCOS groups**

	**P (n = 785)**	**non P (n = 297)**	***P***
BMI (kg/m^2^)	23.87 ± 4.85^a^	22.30 ± 3.27	<0.001
E_2_ (pmol/L)	254.81 ± 169.20^a^	219.73 ± 166.32	0.002
T (nmol/L)	2.60 ± 1.58^a^	1.20 ± 0.70	<0.001
E_2_/T	0.10 (0.06-0.16)^a^	0.16 (0.10-0.29)	<0.001
FSH (mIU/L)	5.98 ± 2.93^a^	6.43 ± 2.17	0.006
LH (mIU/L)	11.90 ± 8.31^a^	5.37 ± 3.80	<0.001
FSH/LH	0.53 (0.36-0.91)^a^	1.41 (1.02-2.05)	<0.001

Data is shown as means ± SD or median and interquartile ranges.

^a^PCOS group compared with non-PCOS group, P < 0.05 suggests significantly different.

P: PCOS group, non P: non-PCOS group, BMI: body mass index, E_2_: estradiol, T: testosterone, FSH: follicle-stimulating hormone, LH: luteinizing hormone.

### Aromatase activity in women with different BMI with or without PCOS

All the three PCOS subgroups manifested lower levels of aromatase activity as compared to the corresponding non-PCOS subgroups (Tables 1 and 2). Furthermore, no significant differences in E_2_/T were observed in both PCOS and non-PCOS subjects who had higher BMI values. However, there were trends demonstrating rising T levels and decreasing E_2_/T and E_2_ levels when BMI values were increased.

**Table 2:** **Biochemical data of the subjects by BMI**

	**BMI ≥ 23 kg/m** ^**2**^			**18.5 kg/m** ^**2**^ **≤ BMI < 23 kg/m** ^**2**^			**BMI < 18.5 kg/m** ^**2**^		
	**P (n = 388)**	**Non P (n = 103)**	***P***	**P (n = 343)**	**Non P (n = 174)**	***P***	**P (n = 54)**	**Non P (n = 20)**	***P***
BMI (kg/m^2^)	27.46 ± 4.39^a,b,d^	25.86 ± 2.66^a,b^	0.001	20.79 ± 1.24^c^	20.73 ± 1.23^c^	0.574	17.54 ± 0.87	17.67 ± 0.95	0.567
E_2_ (pmol/L)	247.95 ± 161.61^d^	213.98 ± 164.51	0.048	258.49 ± 174.83^d^	222.07 ± 166.43	0.023	280.72 ± 185.52	228.93 ± 182.02	0.287
T (nmol/L)	2.60 ± 1.66^d^	1.23 ± 0.64^b^	<0.001	2.57 ± 1.45^d^	1.21 ± 0.74^c^	<0.001	2.81 ± 1.80^d^	0.86 ± 0.53	<0.001
E_2_/T	0.10 (0.06-0.15)^d^	0.15 (0.09-0.24)	0.001	0.10 (0.06-0.16)^d^	0.17 (0.10-0.33)	<0.001	0.09 (0.06-0.16)^d^	0.20 (0.11-0.65)	<0.001
FSH (mIU/L)	5.74 ± 2.80^a,d^	6.40 ± 2.47	0.027	6.19 ± 2.79^d^	6.44 ± 2.06	0.045	6.40 ± 4.29	6.50 ± 1.46	0.916
LH (mIU/L)	10.16 ± 7.24^a,b,d^	5.22 ± 3.82	<0.001	13.24 ± 8.94^b,d^	5.39 ± 3.75	<0.001	15.90 ± 8.52^d^	5.98 ± 4.15	<0.001
FSH/LH	0.63 (0.42-1.03)^b,d^	1.46 (1.00-2.11)	<0.001	0.47 (0.34-0.79)^d^	1.40 (1.05-2.01)	<0.001	0.36 (0.36-0.51)^d^	1.38 (0.87-1.75)	<0.001

Data is shown as means ± SD or median and interquartile ranges.

*P*: PCOS group compared with non-PCOS group.

^a^BMI ≥ 23 kg/m^2^ subgroup compared with 18.5 ≤ BMI < 23 kg/m^2^ subgroup, P < 0.05 means significantly different.

^b^BMI ≥ 23 kg/m^2^ subgroup compared with BMI < 18.5 kg/m^2^ subgroup, P < 0.05 means significantly different.

^c^18.5 ≤ BMI < 23 kg/m^2^ subgroup compared with BMI < 18.5 kg/m^2^ subgroup, P < 0.05 means significantly different.

^d^PCOS/non PCOS subgroups compared in the same BMI degree, P < 0.05 means significantly different.

P: PCOS group, non P: non PCOS group, BMI: body mass index, E2: estradiol, T: testosterone, FSH: follicle-stimulating hormone, LH: luteinizing hormone.

### Aromatase activity in PCOS patients with different E_2_ levels

Higher E_2_ levels correlated with a relatively enhanced E_2_/T as well as T and LH levels but reduced BMI, FSH and FSH/LH levels in women with PCOS (Table 3).

**Table 3:** **Biochemical data of PCOS patients by E**_**2**_ **levels**

	**P (n = 785)**			
	**E**_**2**_ **> 293.6 pmol/L (n = 233)**	**146.8 ≤ E**_**2**_ **≤ 293.6 pmol/L (n = 348)**	**E**_**2**_ **< 146.8 pmol/L (n = 204)**	***P***
BMI (kg/m^2^)	23.32 ± 4.58^b^	23.92 ± 4.90	24.40 ± 5.03	0.034
E_2_ (pmol/L)	455.73 ± 169.42^a,b^	212.23 ± 40.01^c^	97.96 ± 29.27	<0.001
T (nmol/L)	2.97 ± 1.53^a,b^	2.59 ± 1.55^c^	2.19 ± 1.59	<0.001
E_2_/T	0.15 (0.11-0.24)^a,b^	0.09 (0.06-0.14)^c^	0.05 (0.03-0.09)	<0.001
FSH (mIU/L)	5.64 ± 3.60^a^	6.21 ± 2.55	5.97 ± 2.63	<0.001
LH (mIU/L)	12.76 ± 9.97^b^	12.12 ± 7.30^c^	10.55 ± 7.71	<0.001
FSH/LH	0.49 (0.33-0.73)^b^	0.52 (0.37-0.86)^c^	0.64 (0.41-1.15)	<0.001

Data is shown as means ± SD or median and interquartile ranges.

*P:* compare in the three subgroups.

^a^E_2_ > 293.6 pmol/L subgroup compared with 146.8 ≤ E_2_ ≤ 293.6 pmol/L subgroup, *P* < 0.05 means significantly different.

^b^E_2_ > 293.6 pmol/L subgroup compared with E_2_ < 146.8 pmol/L subgroup, *P* < 0.05 means significantly different.

^c^146.8 ≤ E_2_ ≤ 293.6 pmol/L subgroup compared with E_2_ < 146.8 pmol/L subgroup, *P* < 0.05 means significantly different.

P: PCOS group, non P: non PCOS group, BMI: body mass index, E2: estradiol, T: testosterone, FSH: follicle stimulating hormone, LH: luteinizing hormone.

### Aromatase activity in PCOS patients with different T levels

Hyperandrogenic PCOS patients had increased E_2_ levels but their aromatase activity was markedly inhibited independent of their BMI values. The gonadotropins FSH and LH were both increased in people with higher T levels. More precisely, a more pronounced increase of LH was observed compared with FSH increase (Table 4).

**Table 4:** **Biochemical data of the PCOS patients by T levels**

	**P (n = 785)**		
	**T ≥ 2.44 nmol/L (n = 364)**	**T < 2.44 nmol/L (n = 421)**	***P***
BMI (kg/m^2^)	23.35 ± 4.16	24.31 ± 5.34	0.076
E_2_ (pmol/L)	289.41 ± 179.69^a^	224.89 ± 153.62	<0.001
T (nmol/L)	3.85 ± 1.46^a^	1.52 ± 0.55	<0.001
E_2_/T	0.07(0.05-0.11)^a^	0.13 (0.09-0.20)	<0.001
FSH (mIU/L)	6.29 ± 2.84^a^	5.71 ± 2.98	0.006
LH (mIU/L)	14.03 ± 9.03^a^	10.06 ± 7.15	<0.001
FSH/LH	0.48 (0.34-0.73)^a^	0.59 (0.39-1.10)	<0.001

Data is shown as means ± SD or median and interquartile ranges.

^a^T ≥ 2.44 nmol/L subgroup compared with T < 2.44 nmol/L subgroup of PCOS, *P* < 0.05 means significantly different.

P: PCOS group, non P: non PCOS group, BMI: body mass index, E2: estradiol, T: testosterone, FSH: follicle-stimulating hormone, LH: luteinizing hormone.

## Discussion

The human aromatase gene contains 10 exons and one of them encodes nine alternative promoters to regulate tissue-specific expression, and the other nine are the protein-coding exons [19]. Aromatase is expressed in specific cell populations of a variety of estrogen-producing tissues, including placenta, ovaries, testes, skin, adipose tissue, bone, brain, and vascular smooth muscle cells [19]. Importantly, aromatase in ovarian granulosa and luteinized granulosa cells plays an important role for women of reproductive age.

In this study, we aimed to discover the association between aromatase activity, obesity and sex hormones in a large, well-described cohort of PCOS patients. However, there is certain controversy regarding the correlation of ovarian aromatase activity with PCOS [16]. The E_2_/T ratio provides important information about aromatase activity because conversion of androgens to estrogens is mediated by CYP19, suggesting that the E_2_/T ratio may be a direct marker of aromatase activity [20]. Based on our data, PCOS is manifested by a typical abnormal hormone pattern where the increase of LH, testosterone, and estradiol is accompanied with reduced levels of FSH, FSH/LH, and E_2_/T. We found a significant decrease of ovarian aromatase activity in women with PCOS as compared to controls which is consistent with previous work [8,16,21]. In the polycystic ovary, theca cells synthesize more androgens than the corresponding cells in a normal ovary. In contrast, granulosa cells in the polycystic ovary have a lower aromatase activity, which results in an imbalance in the production of estrogen and androgen. An earlier research by Soderlund and co-workers found no gross deletions or insertions after PCR amplification of the nine exons of the P450 arom gene from the peripheral blood leukocytes of 25 PCOS patients [22]. But this cannot preclude the importance of an aromatase disorder in the etiology of PCOS, as there may exist causative mutations in the untranslated regions or within introns.

There is evidence that obesity, particularly abdominal obesity, exacerbates both the clinical and endocrine features of PCOS [23] which demonstrates significantly more serious insulin resistance in these individuals than normal-weight counterparts [24]. Although obesity is not included in the diagnostic criteria for PCOS, 35% to 80% of PCOS women, depending on the setting of the study and the ethnic characteristics of the patients, are commonly overweight (BMI above 25 kg/m^2^) or obese (BMI above 30 kg/m^2^) [25]. Our findings associated higher BMI with PCOS but no concomitant change of E_2_/T was observed. It is reported that estrogen has the capacity to favorably regulate body composition and glucose homeostasis to prevent diet-induced obesity [26].

Aromatase expression in the ovarian follicle is also responsible for the cyclic changes in serum estradiol levels and the modulation of the structure and function of the female reproductive tract and is essential for the survival, fertilization and implantation of oocytes. PCOS promotes a hyperestrogenic state. In this research, PCOS with high estradiol levels led to more serious hyperandrogenism but with relatively elevated levels of E_2_/T. This is consistent with a previous study showing that higher estradiol levels are caused by increased RNA expression of granulosa cell aromatase and its activity [27].

Nevertheless, high testosterone levels of PCOS inhibited aromatase activity. We hypothesize that androgen can dose-dependently affect aromatase activity directly, or indirectly by regulating other factors such as E_2_ and LH. One report suggests that early exposure of females to androgen induces sex-specific organizational changes of aromatase expression in the preoptic area [28].

Hyperinsulinemia and insulin resistance play a role in the pathogenesis of PCOS. Analyzing existing studies, the relationship between insulin and activity of cytochrome P450 family has been explored. La Marca gave a direct demonstration that decreasing insulin with metformin led to a reduction in stimulated ovarian P450c17α activity in PCOS and Nestler JE’s study showed the similar result in lean PCOS [29,30]. Whether P450 aromatase activity may be dependent on insulin resistance will be investigated in our follow-up work.

PCOS is a common ovulatory disorder in young women, which affects 5-10% of the population and results in infertility due to anovulation [31]. Although the pathogenesis of PCOS is still unclear, the role of hyperandrogenism in the pathophysiology of PCOS has been established but not clearly. Oral contraceptives, such as ethinylestradiol and cyproterone acetate tablets, drospirenone and ethinylestradiol tablets, have been used in the clinic to reduce hyperandrogen and to suppress follicular development. Letrozole, an aromatase inhibitor, can induce ovulation in PCOS so that a normal serum androgen level can be maintained by blocking the early low estrogen negative feedback. Generation and metabolism of androgen is directly related to aromatase activity. Along this line, aromatase-agonist-like-drugs perhaps can directly induce follicular development and shorten the treatment course of PCOS women irrespective of the original hyperandrogen state.

### Study limitation

We checked controls for PCOS features (PCOM, hyperandrogenism, oligo-anovulation) in this study. Testosterone was the only androgen that was measured in our study and other androgens such as androstenedione and dehydroepiandrosterone sulfate do not be analyzed because of the limitation of research funding.

## Conclusion

Taken together, our results showed that ovarian aromatase activity in PCOS was decreased which was independent of BMI. Hyperestrogen promoted ovarian aromatase activity which could be inhibited by hyperandrogenism in a dose-dependent manner demonstrating a complex biphasic correlation.

In both normal women and PCOS patients, estrogen levels negatively correlated to BMI. Aromatase, the master convertor of androgen to estrogen, can regulate the estradiol-to-testosterone ratio and thereby regulates BMI. Thus, enhancing aromatase activity may become an optimized strategy for developing therapies for PCOS women, especially those with obesity.
